# Estimation of split renal function on PET using the SSTR-targeting radioligands [^18^F]SiTATE, [^68^Ga]Ga-DOTA-TATE and [^68^Ga]Ga-DOTA-TOC in a theranostic setting

**DOI:** 10.1007/s00259-026-07909-z

**Published:** 2026-05-16

**Authors:** Maximilian Tiling, Lena M. Unterrainer, Sophie C. Siegmund, Josef Zahner, Zachary Ells, Franz J. Gildehaus, Gabriel T. Sheikh, Marcus Unterrainer, Konrad Klimek, Mathias J. Zacherl, Guido Böning, Rudolf A. Werner, Astrid Delker, Adrien Holzgreve

**Affiliations:** 1 Department of Nuclear Medicine, LMU University Hospital, LMU Medizin, LMU Munich, Munich, Germany; 2 Department of Nuclear Medicine and Theranostics, Ahmanson Translational Theranostics Division, David Geffen School of Medicine at UCLA, University of California Los Angeles, Los Angeles, CA USA; 3 Bavarian Cancer Research Center (BZKF), partner site Munich, Munich, Germany; 4 DIE RADIOLOGIE, Munich, Germany; 5 Division of Nuclear Medicine and Molecular Imaging, The Russell H Morgan Department of Radiology and Radiological Sciences, Johns Hopkins School of Medicine, Baltimore, MD USA; 6 Department of Nuclear Medicine, LMU University Hospital, LMU Medizin, LMU Munich, Marchioninistr. 15, 81377 Munich, Germany

**Keywords:** SSTR-PET/CT, Neuroendocrine neoplasms (NEN), Renal function, [^177^Lu]Lu-DOTA-TATE therapy, Dosimetry, [^99m^Tc]Tc-MAG3 scintigraphy

## Abstract

**Purpose:**

SSTR-PET is routinely used to select patients with neuroendocrine neoplasms (NEN) for peptide receptor radionuclide therapy (PRRT). As the kidneys are organs at risk, this study evaluated whether renal uptake on pre-therapeutic SSTR-PET/CT reflects split or global renal function and correlates with renal absorbed dose during [^177^Lu]Lu-DOTA-TATE therapy.

**Methods:**

In this retrospective study, 85 NEN patients treated with [^177^Lu]Lu-DOTA-TATE between 2013 and 2023 were included. All underwent SSTR-PET/CT using [^18^F]SiTATE, [^68^Ga]Ga-DOTA-TATE, or [^68^Ga]Ga-DOTA-TOC plus serum kidney function tests and [^99m^Tc]Tc-MAG3 scintigraphy. Renal PET uptake was compared with serum and scintigraphic kidney parameters. In 30 patients, renal absorbed dose was estimated using post-treatment SPECT/CT.

**Results:**

Renal uptake on SSTR-PET/CT moderately correlated with split renal function assessed by [^99m^Tc]Tc-MAG3 across all tracers, particularly using SUV_mean_ ([^18^F]SiTATE (*r* = 0.462; *p* = 0.010); [^68^Ga]Ga-DOTA-TATE (*r* = 0.515; *p* = 0.004); [^68^Ga]Ga-DOTA-TOC (*r* = 0.546; *p* = 0.004)). No relevant correlation was observed between total renal PET uptake and tubular extraction rate, eGFR, or serum creatinine. Renal PET uptake showed moderate correlations with mean absorbed doses in selected tracer cohorts ([^18^F]SiTATE; *r* = 0.585; *p* = 0.076; [^68^Ga]Ga-DOTA-TATE; *r* = 0.649; *p* = 0.049). A strong negative correlation between TER and absorbed dose was seen only for [^18^F]SiTATE (*r* = − 0.768; *p* = 0.010).

**Conclusion:**

SSTR-PET/CT showed moderate potential to estimate pre-therapeutic split renal function and renal absorbed dose. However, global renal function could not be reliably estimated using PET/CT. Larger studies are warranted to validate PET/CT-based kidney assessment for individualized PRRT planning.

**Supplementary Information:**

The online version contains supplementary material available at 10.1007/s00259-026-07909-z.

## Background/introduction

Neuroendocrine neoplasms (NENs) are a relatively rare group of epithelial tumors in the general population, but with an increasing incidence from 1.09 in 1973 to 3.51 per 100,000 in 2012 worldwide, and a 4.88-fold increase from 1.52 to 7.41 cases per 100,000 in the United States [1]. NENs can express somatostatin receptors (SSTRs) on the cell membrane, enabling specific binding of radiolabeled SSTR analogs [2, 3]. SSTR expression enables functional imaging and targeted therapy (Peptide Receptor Radionuclide Therapy - PRRT), which represents a systemic treatment option for inoperable or metastatic NENs [4]. The baseline SSTR PET/CT provides essential information on patient eligibility for PRRT based on the target expression and candidates for PRRT should exhibit strong SSTR expression [5]. In addition to tumor cells, SSTRs are physiologically expressed in renal tissue, leading to a high renal uptake and accumulation of SSTR ligands, especially in the proximal tubular cells, the glomeruli, and the vasa recta [5, 6]. The renal uptake of the radioligand and its excretion predominantly by the kidneys can result in nephrotoxicity. Primarily because of the highly radiation-sensitive glomeruli, the kidneys are organs at risk in the context of [^177^Lu]Lu-DOTA-TATE therapy. To mitigate this risk, a pre-therapeutic protective amino acid solution containing L-lysine and L-arginine is administered to reduce tubular reabsorption and renal radiation exposure [7]. 

According to EANM guidelines, renal scintigraphy is recommended before PRRT to assess renal function and evaluate the potential nephrotoxic effect of [^177^Lu]Lu-DOTA-TATE therapy, particularly in patients with risk factors for renal toxicity or with compromised renal function [8]. This is often part of the standard protocol in Europe. By contrast, a purely PET/CT-based quantification of split renal function has recently been proposed using renal [^18^F]PSMA-1007 tracer uptake in patients with prostate cancer [9] which may spare patients the additional kidney function scan and would enable pre-therapeutic screening in a one-stop-shop approach. It is unknown whether this innovative concept could be translated from PSMA PET in prostate cancer patients to a similar theranostic setting in patients with NEN.

Therefore, we investigated in a theranostic setting whether it is feasible to estimate renal function using [^18^F]SiTATE, [^68^Ga]Ga-DOTA-TATE, or [^68^Ga]Ga-DOTA-TOC uptake on PET, and whether pre-therapeutic renal tracer uptake on SSTR-PET correlates with the renal absorbed dose of [^177^Lu]Lu-DOTA-TATE.

## Methods

### Study design and patients

A total of 85 patients with NEN treated with [^177^Lu]Lu-DOTA-TATE between 2013 and 2023 at the Department of Nuclear Medicine, LMU University Hospital in Munich, Germany, were included in this retrospective study, with an intended therapeutic activity of 7.4 GBq per treatment cycle. Prior to their first cycle, all patients had undergone blood sampling, PET/CT with an SSTR-targeted radioligand (*n* = 30 [^18^F]SiTATE; *n* = 29 [^68^Ga]Ga-DOTA-TATE; *n* = 26 [^68^Ga]Ga-DOTA-TOC), and [^99m^Tc]Tc-MAG3 scintigraphy (“renal scan”). Renal radioligand uptake in SSTR-PET/CT was assessed in all 85 patients and compared to serum kidney function parameters (creatinine, eGFR) and to [^99m^Tc]Tc-MAG3 scintigraphy–derived parameters, including tubular extraction rate (TER) and split renal function (%). In 30 of 85 patients (*n* = 10 [^18^F]SiTATE; *n* = 10 [^68^Ga]Ga-DOTA-TATE; *n* = 10 [^68^Ga]Ga-DOTA-TOC), post-treatment SPECT/CT was available and utilized for estimation of renal absorbed dose after [^177^Lu]Lu-DOTA-TATE treatment. Results of pre-therapeutic SSTR-PET/CT, serum kidney function parameters, and [^99m^Tc]Tc-MAG3 scintigraphy were correlated to renal absorbed doses based on post-treatment SPECT/CT.

The patients gave written consent for all examinations and the local ethics committee granted approval for the retrospective scientific analysis (#24–0982).

### SSTR PET/CT

PET/CT scans were acquired on a Biograph mCT Flow 20-4R PET/CT (Siemens Healthineers, Erlangen, Germany) and a Biograph 64 TruePoint PET/CT (Siemens Healthineers, Erlangen, Germany). [^18^F]SiTATE, [^68^Ga]Ga-DOTA-TATE and [^68^Ga]Ga-DOTA-TOC were prepared according to previously described protocols [10, 11]. To assess potential scanner-related variability, additional inter-scanner analyses were conducted. Detailed scanner distribution and analysis results are provided in the Supplementary Materials (Online Resource 1, Tables S1 and S2). PET acquisition time was 20 min and started 60–90 min following weight-adapted injection of 210 ± 47 MBq [^18^F]SiTATE, 218 ± 35 MBq [^68^Ga]Ga-DOTA-TATE, or 221 ± 40 MBq [^68^Ga]Ga-DOTA-TOC. When feasible, 20 mg of furosemide were administered concurrently with the tracer to accelerate renal excretion. On the Siemens Biograph mCT, images were reconstructed using TrueX with point-spread-function modelling and time-of-flight (TOF). On the Siemens Biograph 64 TruePoint, TrueX reconstruction without TOF was applied. All reconstructions used a 200 × 200 matrix, 2 iterations with 21 subsets, and a 4-mm Gaussian post-filter. The average time interval between SSTR-PET/CT and therapy was 52 ± 29 days (range: 7–138 days).

### [^99m^Tc]Tc-MAG3 scintigraphy

Renal scintigraphy with ^99m^Tc-labeled MAG3 was performed on a dual-headed gamma camera (Siemens E.Cam; Siemens; Erlangen, Germany) equipped with a low-energy, high-resolution collimator [12]. Imaging acquisition started with bolus injection of 98 ± 5 MBq [^99m^Tc]Tc-MAG3. 15 min post-injection, 20 mg of furosemide were administered for further exploration of delayed excretion, if necessary. The tubular extraction rate (TER) was assessed from measured activity concentrations in blood samples using the single sample method of Bubeck [13, 14]. Split renal function was obtained on the basis of the cumulative relative activity content observed between 60 and 120 s following the application.

The mean interval between [^99m^Tc]Tc-MAG3 scintigraphy and PET/CT was 45 ± 30 days (range: 0–137 days), and the average interval between [^99m^Tc]Tc-MAG3 scintigraphy and therapy was 7 ± 13 days (range: 0–60 days).

An obstruction of the urinary tract system was excluded.

### Creatinine and eGFR

The serum creatinine values were obtained from blood samples on the day of the [^99m^Tc]Tc-MAG3 examination, if available (*n* = 63), or the closest previous/subsequent serum creatinine values were utilized (*n* = 22, mean 11 ± 7 days apart).

eGFR was calculated using the CKD-EPI creatinine equation based on serum creatinine, age, and sex at the time of blood sampling [15]. As all patients in our cohort were white, no race-based adjustment was applied.

### Post-treatment SPECT/CT

SPECT/CT acquisitions were performed at 24, 48, and 72 h post-injection on a Siemens Symbia T2 SPECT/CT system, while SPECT-only acquisitions were obtained on a Siemens E.CAM SPECT system (Siemens Healthineers, Erlangen, Germany) following the first cycle of [^177^Lu]Lu-DOTA-TATE treatment. Detailed acquisition parameters are provided in the previously described protocol [16]; briefly, each scan lasted 15 min. CT-based attenuation correction was performed during quantitative SPECT reconstruction. In 11 patients in whom no CT was acquired along with the SPECT acquisition, the diagnostic CT acquired during PET/CT imaging was used instead. SPECT images were reconstructed using Hermes Hybrid Recon-Oncology 4.0 software (Hermes Medical Solutions, Stockholm, Sweden) in accordance with the departmental standard protocol for ^177^Lu SPECT reconstruction [16], which includes CT-based attenuation correction, Monte Carlo-based scatter correction, and resolution modeling.

### Assessment of renal function on PET

**Fig. 1:**
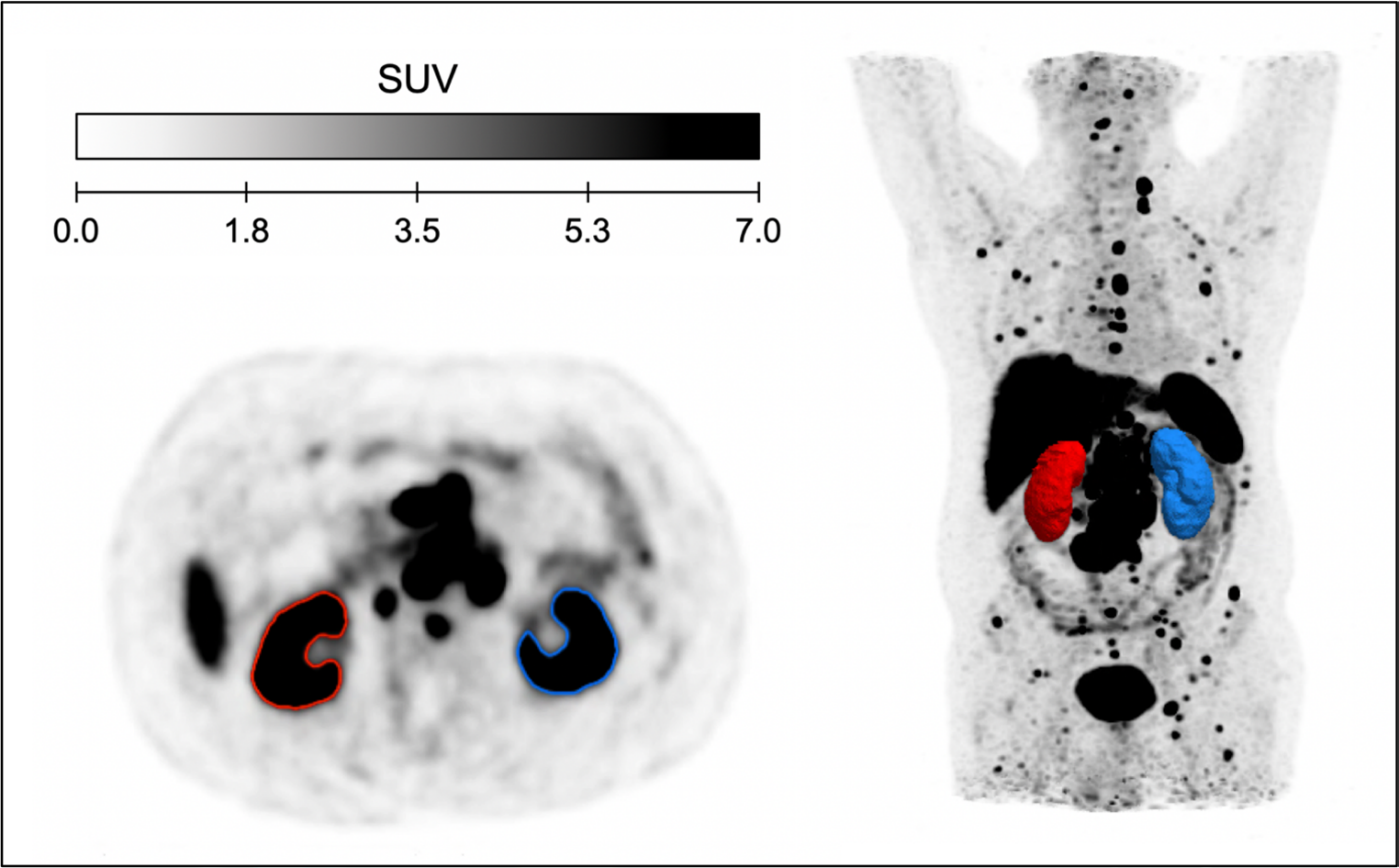
Example of kidney segmentation in a case of [^18^F]SiTATE-PET/CT. SUV: Standardized uptake value. Right kidney volume (red): 184 ml; SUV_max_ 20.0; SUV_mean_ 10.9. Left kidney volume (blue): 195 ml; SUV_max_ 18.4; SUV_mean_ 10.2

The software Affinity Hermia Multimodality Viewer (Hermes Medical Solutions, Stockholm, Sweden) was used to obtain the volume and the standardized uptake values (SUVs) of the kidneys on PET (Fig. 1). Kidney volumes of interest (VOIs) were semi-automatically delineated using a percentage-of-SUV_max_ threshold approach, conceptually adapted from previously published PET-based renal segmentation methodology [9]. Tracer-specific thresholds were defined prior to full cohort analysis based on visual assessment of representative cases and applied consistently within each tracer cohort ([^18^F]SiTATE: 25% of SUV_max_; [^68^Ga]Ga-DOTA-TATE: 32.5% of SUV_max_; [^68^Ga]Ga-DOTA-TOC: 25% of SUV_max_). Fused PET/CT images were used for anatomical guidance. Tracer accumulation within the renal pelvis and ureter was systematically excluded. Urinary activity was identified on co-registered PET/CT images and removed by manual contour refinement, ensuring restriction of VOIs to renal parenchyma. All segmentations were visually inspected and manually adjusted when necessary. Intra-observer reproducibility was assessed in five patients comprising ten kidneys. Each kidney was segmented five times by the same reader at different time points while blinded to previous measurements. Reproducibility was evaluated using the intraclass correlation coefficient (ICC) calculated with a two-way mixed-effects model with absolute agreement for single measurements.

The methodology employed for the calculation of unilateral and bilateral tracer uptake is outlined below. The formulae utilized are based on previously published literature [9].

To determine global renal function, threshold-based kidney volume and SUV_mean_ were employed for the assessment of bilateral renal uptake index (RUI). RUI was calculated as renal volume x SUV_mean_ and used as an SUV-weighted volumetric index. The bilateral RUI was then correlated with the tubular extraction rate (TER, in mL/min) from [^99m^Tc]Tc-MAG3 scintigraphy.

$$\\\begin{array}{c}RUI\;=\\\;(left\;volume\;\times\;left\;SUVmean)\;+\\\;(right\;volume\;\times\;right\;SUVmean)\end{array}$$

Additionally, the pre-therapeutic RUIs from SSTR-PET/CT were correlated with creatinine (in mg/dL) and estimated glomerular filtration rate (eGFR, in mL/min/1.73 m²).

For the assessment of split renal function (SRF), two distinct approaches were used. The first approach was based on a threshold-based analysis of kidney volume and SUV_mean_ and displays the relative tracer uptake of the single kidney (i.e. the *left* kidney in the formula below, exemplarily). It is designated as SRF-SUV_mean_.

$$\\\begin{array}{c}SRF,\;SUVmean=\\\frac{(left\;volume\;\times\;left\;SUVmean)}{(RUI)}\end{array}$$

The second approach was based solely on renal SUV_max_ and is denoted as SRF-SUV_max_.

$$\\\begin{array}{c}SRF,\;SUVmax=\\\frac{left\;SUVmax}{(left\;SUVmax\;+\;right\;SUVmax)}\end{array}$$

Subsequently, the resulting SRF-SUV_mean_ and SRF-SUV_max_ values were correlated with split renal function derived from [^99m^Tc]Tc-MAG3 scintigraphy (SRF_MAG3_). Split renal function was calculated for both kidneys. For correlation analysis and graphical presentation, one side (left kidney) is shown, as left and right renal function values are complementary.

### Kidney dosimetry

Dosimetry was performed using the MIM SurePlan™ MRT version 7.3.4 software (MIM Software, Cleveland, OH, USA). The kidneys were manually delineated on the reference CT scan used for reconstruction of the first SPECT acquisition time point. A time-integrated activity curve was extracted for each contour. Absorbed dose calculations are based on a monoexponential curve fit and local density scaling.

For correlation analyses, the dosimetric endpoint was defined as renal absorbed dose per administered activity (Gy/GBq), allowing standardized comparison across patients independent of injected activity. The renal absorbed dose per administered activity was correlated with pre-therapeutic tracer uptake on SSTR-PET/CT and the pre-therapeutic tubular extraction rate (TER) derived from [^99m^Tc]Tc-MAG3 scintigraphy.

### Statistical analysis

Statistical analysis was performed using GraphPad Prism version 8.4.3 (GraphPad Software, San Diego, CA, USA) and Microsoft Excel 2016 software (Microsoft Corporation, Redmond, WA, USA). The normal distribution of the data was evaluated using the Shapiro–Wilk test. A p-value < 0.05 (two-sided) was considered statistically significant. For analyses in which both variables were normally distributed, Pearson’s correlation coefficient was applied. In cases where at least one variable deviated from normal distribution, Spearman’s rank correlation coefficient was used. Correlation coefficients are reported together with their corresponding 95% confidence intervals. A correlation coefficient of 0.00–00.09 was defined as negligible, 0.10–0.39 as weak, 0.40–0.69 as moderate, 0.70–0.89 as strong, and 0.90–1.00 as a very strong linear correlation [17].

The primary endpoint of this study was the correlation between PET-derived SRF-SUV_mean_ and split renal function assessed by [^99m^Tc]Tc-MAG3 scintigraphy (SRF_MAG3_) within each tracer cohort. All other analyses, including correlations with global renal function parameters (TER, eGFR, creatinine) and dosimetry-based endpoints, were considered exploratory. Given the hypothesis-generating nature of this retrospective study, no formal correction for multiple testing was applied.

A sensitivity analysis was performed for the primary endpoint by stratifying patients according to diuretic administration. In addition, a sensitivity analysis for the primary endpoint was performed by restricting analyses to patients with a PET–MAG3 interval ≤ 30 days to evaluate the influence of examination timing.

## Results

This study included 85 patients with neuroendocrine neoplasms (NEN) who underwent treatment with [^177^Lu]Lu-DOTA-TATE. An overview of patient characteristics is given in Table 1.

**Table 1.**
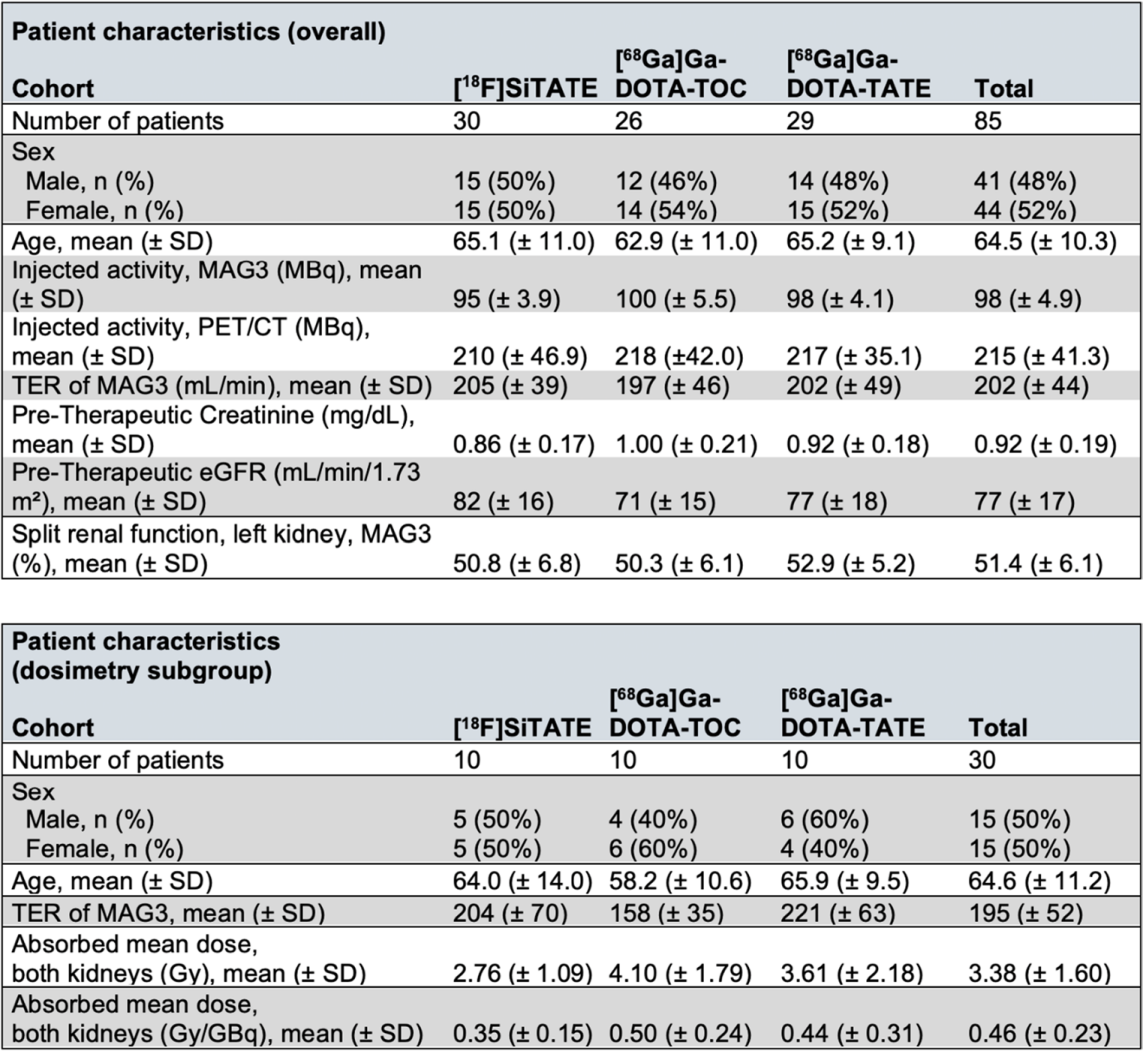
Patient characteristics

### Split renal function

Correlation analysis of [^99m^Tc]Tc-MAG3 scintigraphy and [^18^F]SiTATE PET/CT showed a moderate, yet significant correlation between SRF_MAG3_ and SRF-SUV_mean_ (Pearson’s *r* = 0.462, 95% CI: 0.122–0.705, *p* = 0.010) and no correlation between SRF_MAG3_ and SRF-SUV_max_ (Spearman’s ρ = −0.014, 95% CI: −0.382–0.358, *p* = 0.941).

In the [^68^Ga]Ga-DOTA-TATE cohort, SRF_MAG3_ and SRF-SUV_mean_ correlated significantly (Pearson’s *r* = 0.515, 95% CI: 0.184–0.742, *p* = 0.004). No correlation was found between SRF_MAG3_ and SRF-SUV_max_ (Spearman’s ρ = 0.057, 95% CI: −0.326–0.424, *p* = 0.770).

**Fig. 2:**
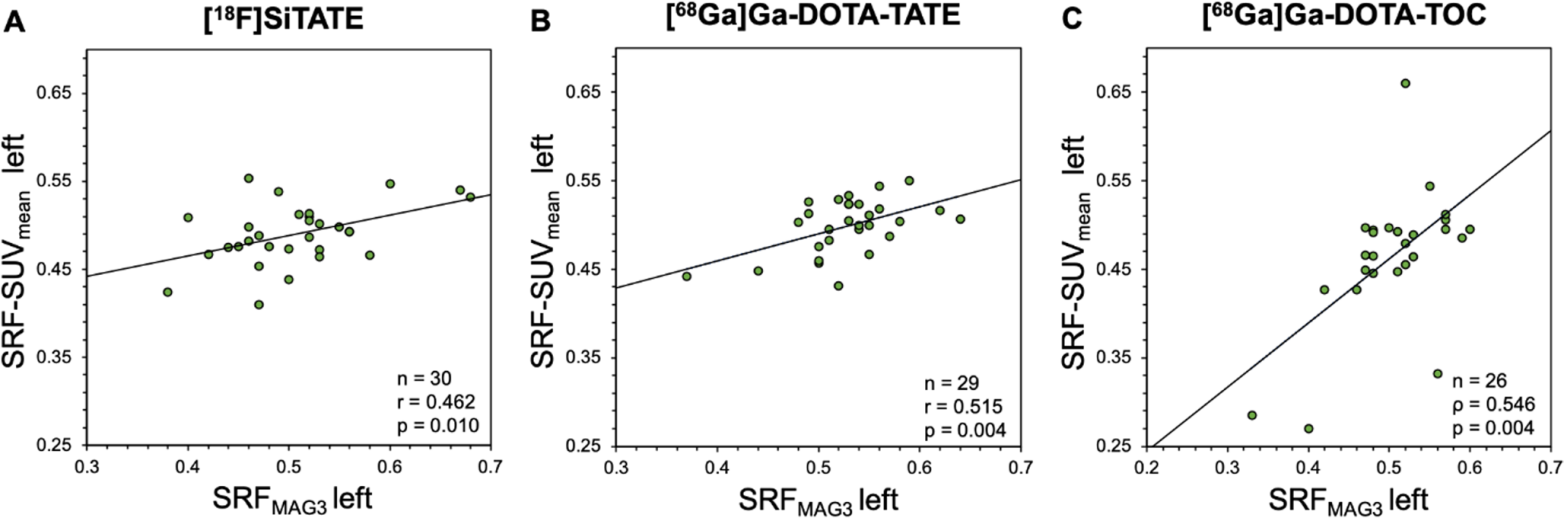
Correlations are shown for the left kidney only; right kidney values are complementary and yield identical correlation results. (**A**) [^18^F]SiTATE: Correlation of left SRF-SUV_mean_ and SRF_MAG3_. (**B**) [^68^Ga]Ga-DOTA-TATE: Correlation of left SRF-SUV_mean_ and SRF_MAG3_. (**C**) [^68^Ga]Ga-DOTA-TOC: Correlation of left SRF-SUV_mean_ and SRF_MAG3_

The [^68^Ga]Ga-DOTA-TOC cohort showed a moderate significant correlation between SRF_MAG3_ and SRF-SUV_mean_ (Spearman’s ρ = 0.546, 95% CI: 0.189–0.776, *p* = 0.004), and a weak positive trend between SRF_MAG3_ and SRF-SUV_max_ (Spearman’s ρ = 0.228, 95% CI: −0.186–0.574, *p* = 0.262). The correlations are reported in Fig. 2.

Correlations between SRF-SUV_mean_ and SRF_MAG3_ remained comparable in direction and magnitude when stratified by diuretic administration, suggesting no systematic influence of furosemide on the primary analysis. Detailed results are provided in Online Resource 1, Table S3. In a sensitivity analysis restricted to patients with a PET–MAG3 interval ≤ 30 days, correlations between SRF-SUV_mean_ and SRF_MAG3_ remained robust and numerically similar to those observed in the full cohorts, indicating that the associations were not materially influenced by the time interval between examinations (see Online Resource 1, Table S4). Intra-observer reproducibility was excellent for renal segmentation parameters based on repeated measurements in a subset of five randomly selected patients (see Online Resource 1, Tables S5, S6, S7, S8).

### Absolute renal function

Renal uptake index (RUI) did not correlate with TER from [^99m^Tc]Tc-MAG3 scintigraphy. [^18^F]SiTATE: (Pearson’s *r* = 0.155, 95% CI: −0.217–0.488, *p* = 0.413), [^68^Ga]Ga-DOTA-TATE: (Pearson’s *r* = − 0.033, 95% CI: −0.395–0.338, *p* = 0.865), [^68^Ga]Ga-DOTA-TOC: (Pearson’s *r* = − 0.035, 95% CI: −0.416–0.358, *p* = 0.867).

**Fig. 3:**
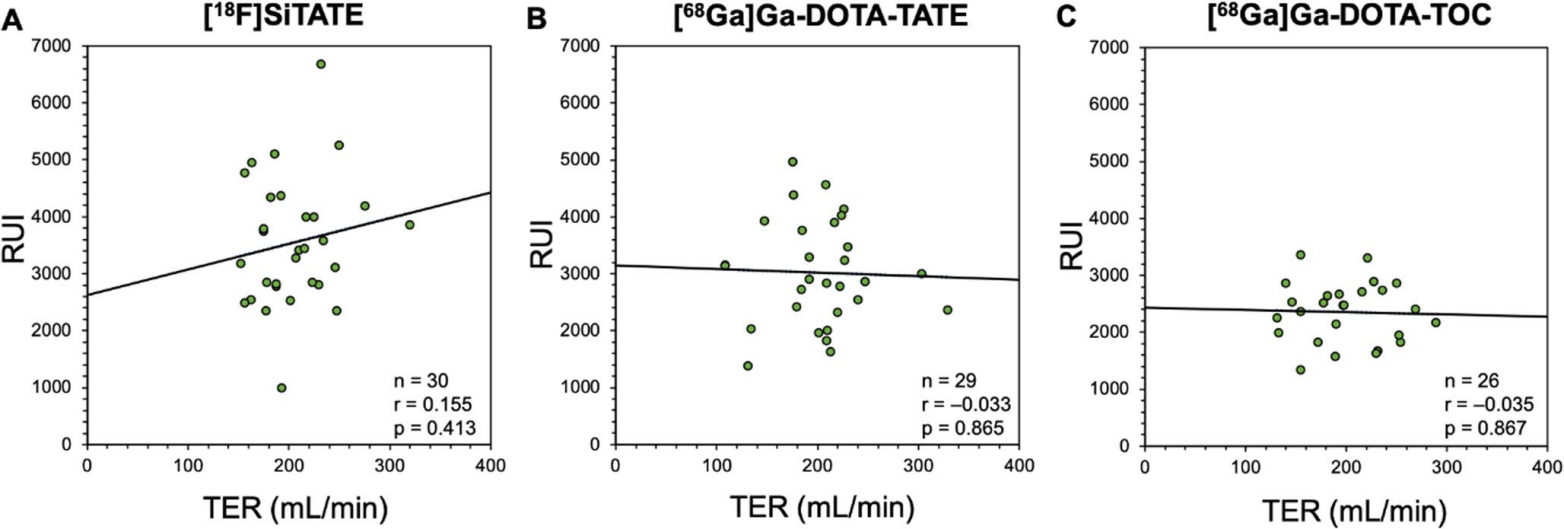
(**A**) [^18^F]SiTATE: Correlation of RUI and TER. (**B**) [^68^Ga]Ga-DOTA-TATE: Correlation of RUI and TER. (**C**) [^68^Ga]Ga-DOTA-TOC: Correlation of RUI and TER

The correlations are displayed in Fig. 3.

The correlation between [^18^F]SiTATE RUI and pretherapeutic serum creatinine showed a weak positive trend (Pearson’s *r* = 0.359, 95% CI: −0.001–0.637, *p* = 0.051). No relevant correlations were found in the [^68^Ga]Ga-DOTA-TATE cohort (Pearson’s *r* = 0.033, 95% CI: −0.338–0.395, *p* = 0.866) and the [^68^Ga]Ga-DOTA-TOC cohort (Pearson’s *r* = 0.064, 95% CI: −0.332–0.440, *p* = 0.756).

No significant association of renal function using RUI and eGFR was revealed in either one of the three cohorts ([^18^F]SiTATE: Pearson’s *r* = − 0.145, 95% CI: −0.480–0.227, *p* = 0.446; [^68^Ga]Ga-DOTA-TATE: Pearson’s *r* = 0.014, 95% CI: −0.356–0.378, *p* = 0.943; [^68^Ga]Ga-DOTA-TOC: Pearson’s *r* = 0.334, 95% CI: −0.061–0.639, *p* = 0.095).

**Fig. 4:**
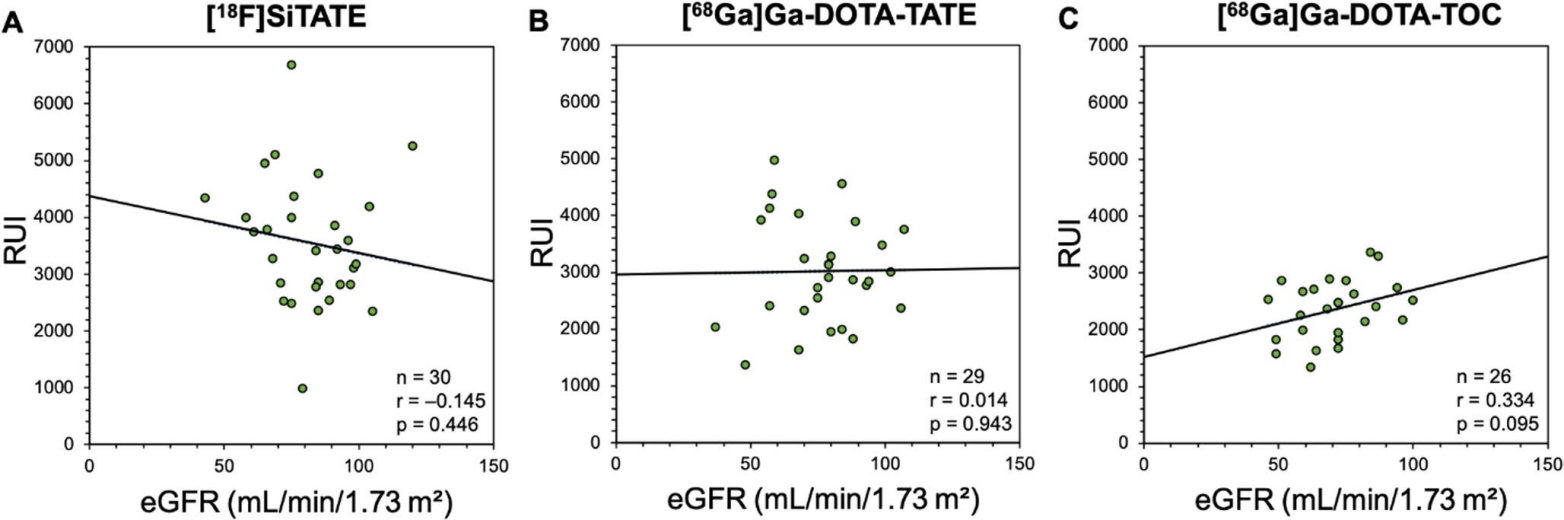
(**A**) [^18^F]SiTATE: Correlation of RUI and eGFR. (**B**) [^68^Ga]Ga-DOTA-TATE: Correlation of RUI and eGFR. (**C**) [^68^Ga]Ga-DOTA-TOC: Correlation of RUI and eGFR

The correlations are shown in Fig. 4.

### Kidney Dosimetry

**Fig. 5:**
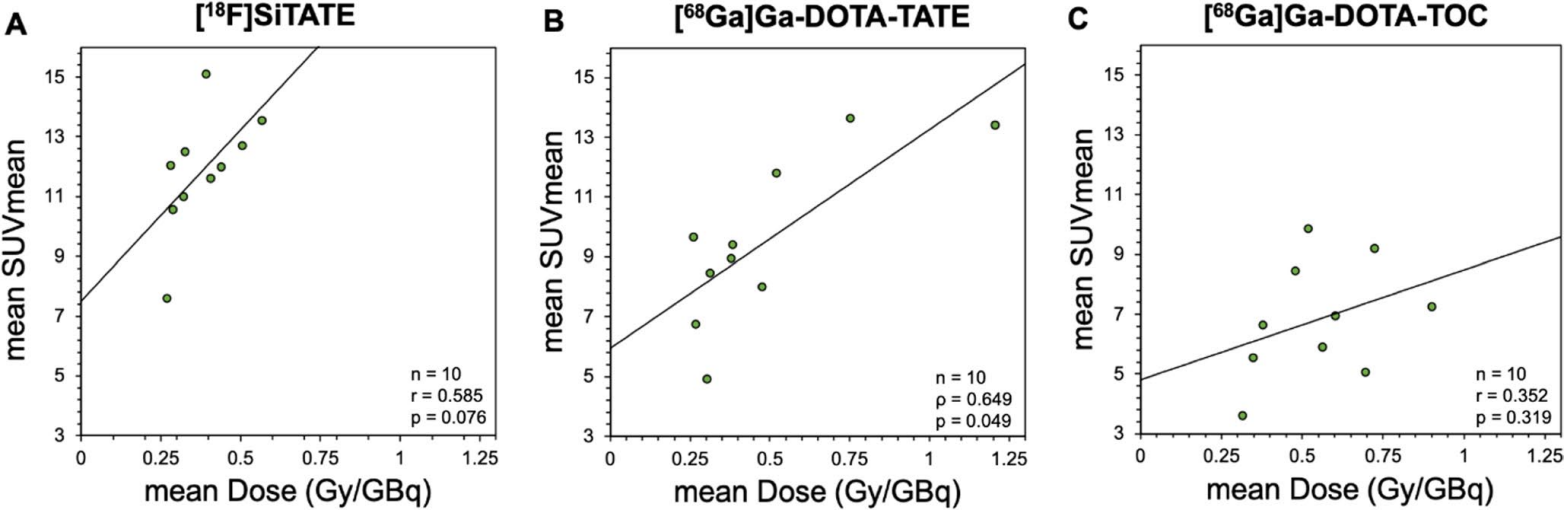
(**A**) [^18^F]SiTATE: Correlation of SUV_mean_ and mean dose (Gy/GBq). (**B**) [^68^Ga]Ga-DOTA-TATE: Correlation of SUV_mean_ and mean dose (Gy/GBq). (**C**) [^68^Ga]Ga-DOTA-TOC: Correlation of SUV_mean_ and mean dose (Gy/GBq)

In the [^18^F]SiTATE cohort, a positive trend was observed between renal uptake in PET/CT (mean SUV_mean_) and mean renal absorbed dose per administered activity of 0.35 ± 0.15 Gy/GBq (Pearson’s *r* = 0.585, 95% CI: −0.071–0.888, *p* = 0.076; Fig. 5). A strong negative and significant correlation was identified between tubular extraction rate (TER) and the renal absorbed dose per administered activity (Pearson’s *r* = − 0.768, 95% CI: −0.942–−0.267, *p* = 0.010).

In the [^68^Ga]Ga-DOTA-TATE cohort, there was a statistically significant positive correlation between renal uptake and mean renal absorbed dose per administered activity of 0.44 ± 0.31 Gy/GBq (Spearman’s ρ = 0.649, 95% CI: 0.031–0.909, *p* = 0.049; Fig. 5) and no relevant correlation between TER and renal absorbed dose (Spearman’s ρ = −0.231, 95% CI: −0.736–0.473, *p* = 0.519).

Two additional positive trends were present in the [^68^Ga]Ga-DOTA-TOC cohort, between renal uptake and mean renal absorbed dose per administered activity of 0.50 ± 0.24 Gy/GBq (Pearson’s *r* = 0.352, 95% CI: −0.357–0.803, *p* = 0.319; Fig. 5) and between TER and renal absorbed dose (Pearson’s *r* = 0.302, 95% CI: −0.405–0.783, *p* = 0.396).

## Discussion

This study evaluates renal tracer uptake of [^18^F]SiTATE, [^68^Ga]Ga-DOTA-TATE, and [^68^Ga]Ga-DOTA-TOC on PET for pre-therapeutic estimation of split renal function and renal absorbed dose in patients with neuroendocrine neoplasms (NENs) in a theranostic setting. Since the kidneys are a dose-limiting organ in peptide receptor radionuclide therapy (PRRT), noninvasive PET-based surrogates of renal function and radiation exposure are of high clinical interest. Our findings show that SSTR-PET/CT provides a promising, though not yet definitive, approach for approximating split renal function and predicting renal radiation absorbed dose. PET-derived parameters were not suitable for assessing global renal function in our cohort.

The statistically significant, moderate correlations between SRF-SUV_mean_ and SRF_MAG3_ in every tracer cohort suggest that PET metrics may serve to approximate relative renal function. This aligns with previous work in PSMA PET/CT imaging, where Rassek et al. demonstrated that renal tracer uptake reflected split renal function in prostate cancer patients [9]. SRF-SUV_max_ instead showed no statistically significant correlation with MAG3-derived split function, suggesting the superiority of SUV_mean_-based analysis for functional approximations in this context. SUV_max_ was included as a potentially simpler and less segmentation-dependent parameter; however, the present findings indicate that focal maximum uptake does not adequately reflect relative renal function.

The renal uptake index (RUI) showed no significant correlation with TER, eGFR, or creatinine, underlining the limitation of PET-based approaches for assessing global renal function. These findings mirror results by Rassek et al., who also reported only weak correlations of bilateral PSMA renal uptake with serum creatinine and TER [9]. Taken together, these results indicate that tracer distribution between kidneys primarily reflects relative perfusion and tubular handling, rather than global renal function.

Dosimetry based on post-treatment SPECT/CT is used to estimate dose delivery to normal organs at risk. Renal PET uptake moderately correlated with renal absorbed doses for [^18^F]SiTATE and [^68^Ga]Ga-DOTA-TATE. This supports the potential use of PET-derived metrics as predictors of renal dose in PRRT [18, 19]. However, in our dosimetry cohort, these findings should be interpreted with caution, as the use of diagnostic CT from PET/CT in a subset of patients may have introduced additional variability in dosimetric calculations. For [^18^F]SiTATE, a strong negative correlation between TER and absorbed dose further indicated that reduced tubular function may increase radiation exposure, consistent with earlier reports by Werner et al. and Svensson et al. [20, 21]. In contrast, no such correlation was observed for [^68^Ga]Ga-DOTA-TATE or [^68^Ga]Ga-DOTA-TOC, suggesting tracer-specific differences in clearance.

These findings imply a mixed mechanism of renal tracer handling. On the one hand, the relative distribution between kidneys is consistent with a flow-dependent process. On the other hand, dosimetry patterns — particularly for [^18^F]SiTATE — point to an additional receptor-mediated component. However, the lack of association with global renal function challenges the idea of predominant SSTR-driven binding. Experimental data from opossum kidney (OK) cells, a well-established proximal tubular cell model, further support the contribution of nonspecific uptake mechanisms such as megalin/cubilin-mediated endocytosis and fluid-phase endocytosis, which together account for a substantial, non-inhibitable fraction of tracer retention [22]. This mechanistic background is clinically relevant, as it explains the prolonged renal retention of [^177^Lu]Lu-DOTA-TATE in PRRT and its role in defining renal absorbed dose, limiting maximum activity, and increasing the risk of nephrotoxicity and myelosuppression [23].

Clinically, these results highlight both the potential and limitations of SSTR-PET/CT. While PET cannot replace comprehensive renal diagnostics — particularly regarding global function — it may provide valuable information on side-separated renal performance and help predict renal radiation dose. In our analysis, renal SUV_mean_ emerged as the central PET-derived parameter, forming the basis of SRF-SUV_mean_ and showing associations with absorbed dose per administered activity. By reflecting side-specific renal performance and anticipated radiation exposure, SUV_mean_ may contribute to more individualized and potentially safer PRRT planning, particularly in patients with asymmetric renal function or borderline renal reserve. In Europe, renal scintigraphy prior to PRRT is considered standard practice, especially for patients at risk of impaired renal function [8], whereas in the U.S., routine scintigraphy is not uniformly recommended [24]. The observed correlation between pre-therapeutic tracer uptake and post-therapeutic renal absorbed dose further suggests that PET-derived metrics could serve as a non-invasive tool to estimate individual renal radiation prior to PRRT. Integrating these measurements may allow earlier identification of clinically relevant renal impairment, support risk stratification, and enable more personalized dose adjustments and treatment planning.

This study has several limitations. It is retrospective in nature and limited by a relatively small sample size, particularly in the dosimetry subgroup. Baseline renal comorbidities were not systematically analyzed in this retrospective cohort and may represent potential confounders influencing renal function parameters and dosimetry estimates. PET/CT scans were acquired on different scanner systems, introducing technical variability. In addition, PET acquisition was performed within a 60–90 min post-injection window, reflecting routine clinical practice. Although prior time–activity analyses [25] have demonstrated relative stability of renal tracer uptake beyond 60 min post-injection, minor variability related to acquisition timing cannot be entirely excluded. Tumor burden may further influence physiologic organ uptake in SSTR imaging through a tumor sink effect and thus represent a potential confounder in SUV-based analyses. In our study, inclusion was restricted to patients undergoing PRRT in a comparable pre-therapeutic setting to reduce heterogeneity in disease stage. Moreover, as our primary endpoint focused on split renal function—an intraindividual comparison between both kidneys—systemic tracer redistribution would be expected to affect both sides similarly and is therefore less likely to substantially bias relative measurements. A sensitivity analysis to assess the impact of CT source on dosimetry could not be performed due to limitations in retrospective data assignment. Therefore, the potential influence of CT source on dosimetric results cannot be excluded and should be considered when interpreting these findings.

## Conclusion

Our findings suggest that SSTR-PET/CT may serve as a tool to estimate split renal function and suggest a correlation between pre-therapeutic tracer uptake and post-therapeutic renal absorbed dose. However, PET-derived metrics did not reliably reflect global renal function. This could provide additional diagnostic value beyond the primary purpose of SSTR-PET/CT in lesion detection and staging, such as functional kidney assessment and contribute to more individualized and safer treatment strategies.

## Supplementary Information

Below is the link to the electronic supplementary material.

Supplementary File 1 (PDF 215 KB)

## Data Availability

The datasets used and/or analyzed during the current study are available from the corresponding author on reasonable request.
