# Supplementary material for: Estimation of split renal function on PET using the SSTR-targeting radioligands [18F]SiTATE, [68Ga]Ga-DOTA-TATE and [68Ga]Ga-DOTA-TOC in a theranostic setting
Source: Eur J Nucl Med Mol Imaging. 2026 May 16;53(9):5491–500. doi: 10.1007/s00259-026-07909-z (PMC13314680; doi:10.1007/s00259-026-07909-z)
Supplement: Supplementary file 1 — (PDF 215 KB) [file 259_2026_7909_MOESM1_ESM.pdf]

SUPPLEMENTARY INFORMATION

European Journal of Nuclear Medicine and Molecular Imaging

Estimation of split renal function on PET using the SSTR-targeting radioligands [^{18}F]SiTATE, [^{68}Ga]Ga-DOTA-TATE and [^{68}Ga]Ga-DOTA-TOC in a theranostic setting

Maximilian Tiling[1]*, Lena M. Unterrainer[1,2,3]*, Sophie C. Siegmund[1,3], Josef Zahner[1], Zachary Ells[1,2], Franz-Josef Gildehaus[1], Gabriel T. Sheikh[1], Marcus Unterrainer[1,4], Konrad Klimek[1], Mathias J. Zacherl[1], Guido Böning[1], Rudolf A. Werner[1,5], Astrid Delker[1], Adrien Holzgreve[1,2]

(1) Department of Nuclear Medicine, LMU University Hospital, LMU Munich, Munich, Germany
(2) Ahmanson Translational Theranostics Division, David Geffen School of Medicine at UCLA, Los Angeles, CA, USA
(3) Bavarian Cancer Research Center (BZKF), partner site Munich, Munich, Germany
(4) DIE RADIOLOGIE, Munich, Germany
(5) Department of Radiology and Radiological Sciences, Division of Nuclear Medicine, The Russell H Morgan, Johns Hopkins School of Medicine, Baltimore, MD, USA

* Shared first authors.

Correspondence:
Maximilian Tiling, Medical Student
Department of Nuclear Medicine, LMU University Hospital, LMU Munich
Marchioninistr. 15, 81377 Munich, Germany
Tel.: +49 89 4400 74646, Fax: +49 89 4400 77646
E-Mail: Maximilian.Tiling@med.uni-muenchen.de
ORCID: 0009-0004-6862-2196
Corresponding author on behalf of Dr. Adrien Holzgreve, MD, MHBA

PET/CT scanner distribution and inter-scanner comparison analyses

Tracer	Biograph mCT Flow 20-4R	Biograph 64 TruePoint
[^{18}F]SiTATE	28	2
[^{68}Ga]Ga-DOTA-TATE	9	20
[^{68}Ga]Ga-DOTA-TOC	26	–

Table S1. Distribution of PET/CT scanners across tracer cohorts.

Scanner	n	SUV_{mean} (mean ± SD)
Biograph mCT Flow 20-4R	9	9.24 ± 3.46
Biograph 64 TruePoint	20	8.97 ± 2.42

Table S2. Inter-scanner comparison of SUV_{mean} values of the left kidneys in the [^{68}Ga]Ga-DOTA-TATE cohort. Group differences were assessed using an unpaired t-test with Welch's correction ($p = 0.83$).

Sensitivity analysis stratified by furosemide administration

Tracer	Furosemide	n	r / ρ	95% CI	p
[^{18}F]SiTATE	Yes	22	r = 0.454	0.041–0.735	0.034
[^{18}F]SiTATE	No	8	r = 0.412	−0.412–0.866	0.310
[^{68}Ga]Ga-DOTA-TATE	Yes	19	r = 0.480	0.033–0.767	0.038
[^{68}Ga]Ga-DOTA-TATE	No	10	r = 0.594	−0.057–0.891	0.070
[^{68}Ga]Ga-DOTA-TOC	Yes	22	ρ = 0.519	0.111–0.777	0.013
[^{68}Ga]Ga-DOTA-TOC	No	4	r = 0.593	−0.856–0.990	0.407

Table S3. Correlation between PET-derived SRF-SUV_{mean} and SRF_{MAG3} stratified by furosemide administration within each tracer cohort. Pearson's r was used for normally distributed variables; Spearman's ρ was used when normality assumptions were not met.

Sensitivity analyses evaluating the influence of the PET–MAG3 interval

Tracer	Cohort	n	r / ρ	95% CI	p
[^{18}F]SiTATE	Full cohort	30	r = 0.462	0.122–0.705	0.010
[^{18}F]SiTATE	≤30 days	12	r = 0.478	−0.132–0.825	0.116
[^{68}Ga]Ga-DOTA-TATE	Full cohort	29	r = 0.515	0.184–0.742	0.004
[^{68}Ga]Ga-DOTA-TATE	≤30 days	8	r = 0.723	0.036–0.946	0.043
[^{68}Ga]Ga-DOTA-TOC	Full cohort	26	ρ = 0.546	0.189–0.776	0.004
[^{68}Ga]Ga-DOTA-TOC	≤30 days	10	ρ = 0.640	0.017–0.905	0.046

Table S4. Sensitivity analysis comparing correlations between PET-derived SRF-SUV_{mean} and SRF_{MAG3} in the full cohort and in patients with a PET–MAG3 interval ≤30 days within each tracer cohort. Pearson's r was used for normally distributed variables; Spearman's ρ was used when normality assumptions were not met.

Intra-observer reproducibility analysis for PET-derived renal segmentation parameters

Parameter	ICC	95% CI	p
Renal volume	0.972	0.927–0.992	<0.001
SUV_{mean}	0.998	0.996–1.000	<0.001
SRF-SUV_{mean}	0.972	0.931–0.992	<0.001

Table S5. Intra-observer reproducibility analysis of PET-derived renal segmentation parameters based on five repeated segmentations in five randomly selected patients (10 kidneys). Intraclass correlation coefficients (ICC) were calculated using a two-way mixed-effects model with absolute agreement for single measurements.

Patient	Kidney	Seg 1 (mL)	Seg 2 (mL)	Seg 3 (mL)	Seg 4 (mL)	Seg 5 (mL)
P1	left	146	145	143	141	140
P1	right	149	146	145	142	145
P2	left	121	122	122	122	122
P2	right	101	101	101	101	101
P3	left	132	126	123	126	131
P3	right	134	133	127	126	132
P4	left	132	132	133	127	126
P4	right	136	136	136	132	135
P5	left	115	114	109	115	114
P5	right	150	147	147	147	147

Table S6. Repeated measurements of renal volume used for intra-observer reproducibility analysis.

Patient	Kidney	Seg 1	Seg 2	Seg 3	Seg 4	Seg 5
P1	left	10.7	10.5	10.7	10.8	10.8
P1	right	10.4	10.0	10.5	10.6	10.5
P2	left	11.8	11.8	11.7	11.8	11.7
P2	right	13.4	13.4	13.4	13.4	13.4
P3	left	5.7	5.9	6.0	5.9	6.2
P3	right	6.1	6.0	6.1	6.1	6.1
P4	left	8.7	8.7	8.7	8.9	8.9
P4	right	9.5	9.6	9.6	9.6	9.6
P5	left	15.1	15.5	15.5	15.5	15.5
P5	right	14.6	14.9	14.9	14.9	14.9

Table S7. Repeated measurements of SUV_{mean} used for intra-observer reproducibility analysis.

Patient	Kidney	Seg 1	Seg 2	Seg 3	Seg 4	Seg 5
P1	left	0.502	0.510	0.501	0.503	0.498
P1	right	0.498	0.490	0.499	0.497	0.502
P2	left	0.513	0.515	0.513	0.515	0.513
P2	right	0.487	0.485	0.487	0.485	0.487
P3	left	0.479	0.482	0.488	0.492	0.502
P3	right	0.521	0.518	0.512	0.508	0.498
P4	left	0.471	0.468	0.470	0.471	0.464
P4	right	0.529	0.532	0.530	0.529	0.536
P5	left	0.442	0.447	0.435	0.449	0.447
P5	right	0.558	0.553	0.565	0.551	0.553

Table S8. Repeated measurements of SRF-SUV_{mean} used for intra-observer reproducibility analysis.
